# Comparison and Fusion of Machine Learning Algorithms for Prospective Validation of PET/CT Radiomic Features Prognostic Value in Stage II-III Non-Small Cell Lung Cancer

**DOI:** 10.3390/diagnostics11040675

**Published:** 2021-04-09

**Authors:** Shima Sepehri, Olena Tankyevych, Taman Upadhaya, Dimitris Visvikis, Mathieu Hatt, Catherine Cheze Le Rest

**Affiliations:** 1 LaTIM, INSERM, UMR 1101, University Brest, 29200 Brest, France; sh_sepehri_ee@yahoo.com (S.S.); tankyevych@gmail.com (O.T.); dimitris@univ-brest.fr (D.V.); catherine.cheze-le-rest@chu-poitiers.fr (C.C.L.R.); 2 Nuclear Medicine Department, CHU Milétrie, 86021 Poitiers, France; tamanupadhaya@gmail.com

**Keywords:** machine learning, prospective study, non-small cell lung cancer, prognosis, radiomics

## Abstract

Machine learning (ML) algorithms for selecting and combining radiomic features into multiparametric prediction models have become popular; however, it has been shown that large variations in performance can be obtained by relying on different approaches. The purpose of this study was to evaluate the potential benefit of combining different algorithms into an improved consensus for the final prediction, as it has been shown in other fields. **Methods:** The evaluation was carried out in the context of the use of radiomics from ^18^F-FDG PET/CT images for predicting outcome in stage II-III Non-Small Cell Lung Cancer. A cohort of 138 patients was exploited for the present analysis. Eighty-seven patients had been previously recruited retrospectively for another study and were used here for training and internal validation. We also used data from prospectively recruited patients (n = 51) for testing. Three different machine learning pipelines relying on embedded feature selection were trained to predict overall survival (OS) as a binary classification: Support Vector machines (SVMs), Random Forests (RFs), and Logistic Regression (LR). Two different clinical endpoints were investigated: median OS or OS shorter than 6 months. The fusion of the three approaches was implemented using two different strategies: majority voting on the binary outputs or averaging of the output probabilities. **Results:** Our results confirm previous findings, highlighting that different ML pipelines select different sets of features and reach different classification performances (accuracy in the testing set ranging between 63% and 67% for median OS, and between 75% and 80% for OS < 6 months). Generating a consensus improved the performance for both endpoints; with the probabilities averaging strategy outperforming the majority voting (accuracy of 78% vs. 71% for median OS and 89 vs. 84% for OS < 6 months). Overall, the performance of these radiomic-based models outperformed the standard clinical staging in both endpoints (accuracy of 58% and 53% accuracy in the testing set for each endpoint). **Conclusion:** Although obtained in a small cohort of patients, our results suggest that a consensus of machine learning algorithms can improve performance in the context of radiomics. The resulting prognostic stratification in the prospective testing cohort is higher than when relying on the clinical stage. This could be of interest for clinical practice as it could help to identify patients with higher risk amongst stage II and III patients, who could benefit from intensified treatment and/or more frequent follow-up after treatment.

## 1. Introduction

Non-small cell lung cancer (NSCLC) remains a deadly disease, despite improvements in diagnosis, staging and treatment. It is the first cause of cancer death for men and the second for women (Bray, et al. 2018). The variability in outcomes is vast, with significant differences between patients depending on the stage of the disease. Staging is indeed one of the major clinical criteria on which physicians rely in order to select a therapeutic strategy (i.e., concomitant or sequential combination of surgery, chemotherapy and radiotherapy) [1]. However, even amongst patients with a similar disease stage, especially for stage II and III, there can be highly variable outcomes (i.e., response to therapy and survival). ^18^F-FDG Positron Emission Tomography / Computed Tomography (PET/CT) has shown its usefulness for NSCLC staging, treatment planning and monitoring [2].

However, these quantitative images are still exploited in clinical routine mostly through visual examination only. Clinical variables, like stage, age, and extent of resection after surgery [3], together with PET and/or CT image-derived engineered features, such as the histogram intensity metrics, tumor geometric shape descriptors or intra-tumor heterogeneity textural features, have been associated with prognosis [4,5]. The extraction of these features through a semi-automated workflow is known today as radiomics [6]. In order to evaluate the predictive and prognostic value of these features, most of earlier radiomic studies relied on inappropriate statistical analysis (e.g., lack of correction for multiple testing, lack of multivariate analysis and external validation sets) that led to overoptimistic results [7]. Later studies relied increasingly on robust machine learning (ML) algorithms in order to build multiparametric models, i.e., combining these radiomic features and other clinical variables to identify a tumor type, correlate with underlying biological information, or predict outcomes [8].

Several studies have highlighted that the performance of the models may vary significantly depending on the chosen ML methodology for feature selection and classification or regression [9,10,11,12]. This is a major issue preventing radiomics-based multiparametric models trained using established ML algorithms from being validated by the community and further translated to clinical practice. These recent studies did not explicitly report on the potential complementary value of the outputs generated by these different approaches or the benefit of generating a consensus. Such a fusion could improve overall performance, and could also increase the reliability of the resulting models, as well as the confidence the community can have about them. In addition, most radiomic studies have been retrospective in nature.

In a previous study, we showed that PET and CT radiomic features can have complementary value over stage alone in NSCLC stage II-III, relying on standard statistical analysis and exploiting a retrospective-only cohort of patients [13].

In the present work, our main objective was to evaluate the potential value of fusing the outputs of different ML algorithms relying on embedded feature selection. In addition, we further investigate the prognostic value of PET/CT radiomic features for stage II-III NSCLC patients in a prospective context.

## 2. Materials and Methods

As previously shown in the retrospectively collected cohort (n = 116), patients with stage I have a much more favorable prognosis compared to these with stages II and III, and radiomic features have more potential to discriminate between patients with better or worse prognosis amongst stage II-III patients [13]. In the present study, we therefore focused on these stage II and III patients.

### 2.1. Patients

A cohort of 138 NSCLC patients with stage II and III treated with curative (chemo) radiotherapy at the CHU (Centre Hospitalier Universitaire) Milétrie in Poitiers, France was exploited in the present work (Table 1). Out of 138 patients, 87 were retrospectively collected and were included in our previous study [13]. Fifty-one additional patients were prospectively collected between 2016 and 2018. Inclusion criteria were: confirmed NSCLC, available FDG PET/CT imaging, stage II or III and (chemo) radiotherapy treatment, and at least one-year follow-up.

All patients provided signed permission for the use of their clinical data for scientific purposes and informed consent for the anonymous publication of data. The “comité de protection des personnes (CPP Ouest III)” (local ethics committee) from the University Hospital of Poitiers approved this study.

### 2.2. PET/CT Imaging Procedure

All patients had an ^18^F-FDG PET/CT acquired as part of their diagnosis and staging procedure prior to treatment, which was exploited in the present work. A Biograph mCT 40 ToF with axial field of view of 21.6 cm (Siemens, Erlangen, Germany) was used, relying on the routine clinical protocol. PET/CT acquisition began after 6 h of fasting and 60 ± 5 min after injection of 2.5 MBq/kg of ^18^F-FDG (421 ± 98 MBq, range 220–695 MBq). Non-contrast-enhanced, non-respiratory-gated (free-breathing) CT images were acquired (120 kVp, Care Dose^®^ current modulation system) with an in-plane resolution of 0.853×0.853 mm^2^ and a 5 mm slice thickness. PET data were acquired using 3.5 min per bed position and images were reconstructed using a CT-based attenuation correction and the OSEM-TrueX-TOF algorithm (with time-of-flight and spatial resolution modelling, three iterations and 21 subsets, 5 mm 3D Gaussian post-filtering, voxel size 4 × 4 × 4 mm^3^).

### 2.3. Image Analysis

**Figure 1:**
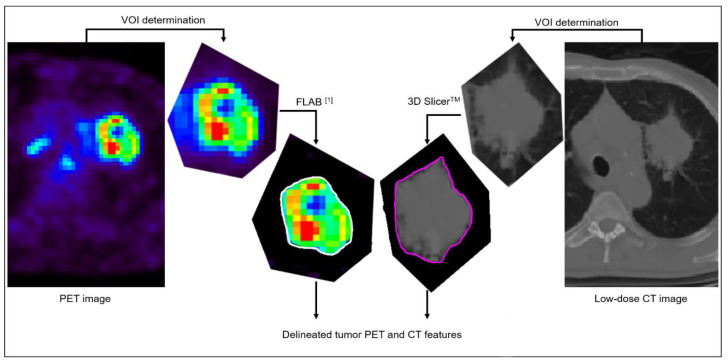
Illustration of the delineation process of PET and CT images of the tumor.

The 87 retrospectively collected datasets were not re-processed and the previously considered volumes of interest [13] were used. A single expert processed the 51 new prospectively collected datasets following the exact same procedure (Figure 1), as follows: primary tumor volumes were characterized independently in FDG PET and in the associated low-dose CT images. Primary tumor volumes were first detected and isolated in a rough volume of interest (VOI) containing the tumor and excluding other surrounding physiological uptakes or anatomical structures as much as possible. Determination of the metabolic tumor volume in the PET image was then automatically performed using the Fuzzy Locally Adaptive Bayesian (FLAB, in-house software, LaTIM, Brest, France) algorithm [14,15] applied to that VOI, whereas anatomical volumes were semi-automatically delineated in low-dose CT images using 3D Slicer^TM^ [16]. All delineations were checked and validated by an expert physician (C. Cheze Le Rest).

After delineation, all volumes of interest for the 138 patients in both PET and CT images were re-processed for radiomic feature extraction, in order to rely on the most up-to-date international Image Biomarker Standardization Initiative (IBSI) recommendations and benchmark values [17].

IBSI-compliant radiomic features, including intensity metrics (n = 10), shape descriptors (n = 14) and textural features (n = 66), were calculated using MIRAS (in-house software, LaTIM, Brest, France). Regarding the 66 2nd- and higher-order textural features, intensities were first discretized using fixed bin width (FBW, with 0.5 SUV for PET and 10 HU for CT) and fixed bin number (FBN, 64 bins) methodologies. Histogram equalization into 64 bins (currently not benchmarked by the IBSI) was also applied in both modalities. Texture matrices were implemented in 3D following the merging strategy (i.e., considering all 13 directions simultaneously).

A total of 222 features (10 + 14 + 66 × 3) were thus extracted from each tumor volume in both PET and CT, leading to 444 image-derived variables for each patient being available as an input to each of the three ML pipelines.

### 2.4. Modeling

The two endpoints investigated were overall survival (OS) below the median or below 6 months, as binary classification problems. The first endpoint corresponds to a balanced classification (i.e., no need to correct for imbalance in the training data), whereas the second endpoint leads to unbalanced datasets but is also more clinically relevant as it corresponds to the identification of a small subset of patients with very poor prognosis that could be offered alternative treatment strategies, such as targeted therapies. In that case, Synthetic Minority Over-sampling Technique (SMOTE) [18] was applied to the training dataset, in order to facilitate learning of the models by generating additional synthetic data points for the minority class, so negative and positive classes are balanced.

The retrospectively recruited patients constituted the training and internal validation set (63%, n = 87). whereas the prospectively recruited patients constituted the testing set (37%, n = 51).

All available clinical variables (gender, clinical stage, smoking history, histology, treatment modality) and radiomic features were entered in the three implemented ML algorithms, namely Support Vector Machines (SVM) with Recursive Feature Elimination (RFE), Random Forests (RF) with Embedded Wrapper (EW) method, and Logistic Regression (LR) with the stepwise method. Hyperparameters for each ML algorithm (e.g., number of trees in RF, depth) were optimized through cross-validation in the training set only. Similarly, our previous investigations suggested that relying on all features (clinical+PET+CT) and including all three discretization techniques led to better performance, so only the results obtained in that configuration are further presented here.

For each ML approach, the best model was selected based on its performance in the training set according to the following criteria: (i) the level of accuracy and balance between sensitivity and specificity and (ii) the number of required features (i.e., for a similar level of performance, a smaller number of features is preferred to minimize the risk of overfitting and for a higher likelihood of generalizability when applied to the testing set).

Finally, in order to generate a consensus of the outputs from the three ML algorithms, we implemented two different strategies: first, a majority-voting rule was applied to the binary outputs of each algorithm. Second, the average of probabilities from each algorithm was calculated before binarization (> or ≤0.5) for the classification.

The performance of each finalized ML algorithm and their fusion (through majority voting or probabilities averaging) was evaluated on the testing set using accuracy (balanced accuracy for the <6 months OS endpoint). Kaplan–Meier (K–M) survival curves were also generated from the classification results.

## 3. Results

At staging, there were 43 stage II patients and 95 stage III patients. With an average follow-up of 40.85 months (range 1.9–95.1 months), the median OS was 14.4 months (range 1.1–50 months). At the last follow-up, 69 patients (50%) had died. 

**Figure 2:**
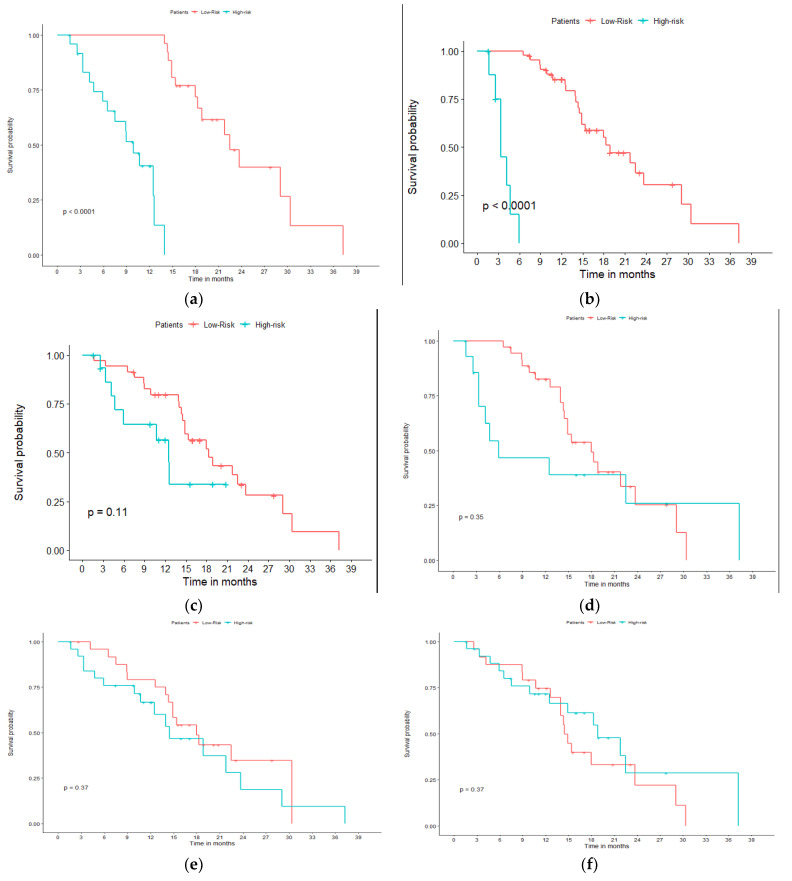
Kaplan–Meier curves obtained in the testing cohort for the two endpoints: (**a**,**c**,**e**) median overall survival (OS) and (**b**,**d**,**f**) OS <6 months. (**a**,**b**) is the ground-truth, (**c**,**d**) is obtained with the groups of patients classified by the probabilities averaging. (**e**,**f**) Stratification achieved with stage II vs. III.

Classification results of each ML approach and their consensus following the two strategies are summarized in Table 2. Figure 2 provides K–M survival curves, obtained using the best models and compared to the clinical staging in the testing set.

Regarding the endpoint of median OS, in the training set, the best model, built by RF with 25 features, reached an accuracy of 89% [95% CI 84–94]. In the testing set, this model obtained only 67% (95% CI 59–75) accuracy. With SVM, the best model, combining 27 features, obtained an accuracy of 100% (95% CI 100–100), but reached only 64% (95% CI 56–72) accuracy when applied to the testing set. Higher accuracy (69% (95% CI 61–77)) could be obtained in the testing set, at the cost of including a much larger number of features, which would likely reduce the performance of the model in external datasets. LR relying on 37 features reached lower accuracy 72% (95% CI 65–79) in the training set compared to RF and SVM, and performed with slightly reduced accuracy in the testing, with 63% (95% CI 55–71) accuracy.

The fusion of the three ML algorithms with majority voting led to a perfect accuracy (100% (95% CI 100–100)) in the training set and slightly increased performance of 71% (95% CI 63–79) (sensitivity 75% (95% CI 68–82), specificity 67% (95% CI 59–75)) in the testing set. Using the average of the probabilities led to even better results of 78% (95% CI 71–85) accuracy (sensitivity 75% (95% CI 68–82), specificity 80% (95% CI 73–87)). This means that, using each of the three algorithms independently correctly identified whether the patients had a survival above or below the median value for 32 (LR) to 34 (RF) of them (out of 51), and this increased to 36 and 40 using majority voting and probability averaging, respectively.

By comparison, the accuracy reached by the standard clinical factor routinely used to stratify patients and determine therapeutic options (stage 2 vs. 3), was only 61% (95% CI 53–69) in the training set and 58% (95% CI 50–66) in the testing set.

Regarding the second endpoint of OS below 6 months, the overall performance of all models was higher, reaching 89% (95% CI 84–94) accuracy with average probabilities of the three ML models, which provided between 75% (95% CI 68–82) and 80% (95% CI 73–87) independently. By comparison, relying on stage alone led to 53% (95% CI 45–61) accuracy only in the testing set.

## 4. Discussion

Although several studies have previously highlighted the differences in terms of performance obtained with different ML algorithms [9,10], most of them did not specifically address the potential benefit of performing a consensus or fusion of their outputs.

Our results, obtained in a prospectively collected testing cohort, seem to confirm our initial findings regarding the prognostic value of PET/CT radiomic features over stage alone [13]. Indeed, although the performance remains moderate, and the models seem to suffer from overfitting (100% accuracy in training but substantially worse result in testing for the median OS endpoint, although predicting OS < 6 months demonstrated a lower decrease in performance between training and testing), prognostic stratification using the radiomic models, especially the fusion result, was nonetheless much better than that relying on clinical stage alone: 78% vs. 58% for median OS and 89% vs 53% for OS < 6 months. The performance was consistently improved for both endpoints when exploiting the consensus of the three ML algorithms, instead of each of them being used separately. Comparing the two strategies, majority voting vs. averaging output probabilities, the latter achieved a higher improvement in both scenarios.

Several other recent studies have investigated the prognostic value of PET/CT radiomic features in NSCLC. Wang, et al. 2017 compared different machine learning and deep learning methods to classify mediastinal lymph node metastasis of NSCLC using ^18^F-FDG PET/CT images [19]. However, they did not consider the fusion of the machine learning techniques before moving to the use of deep learning methods. Mi, et al. 2015 proposed a novel wrapper feature selection algorithm to overcome the small sample size problem of datasets [20]. This new wrapper technique showed promising results, in the same range of accuracy as ours for an SVM classifier. Since their Hierarchical Forward Selection wrapper helps to have better results for small data, the use of fusion might be of interest to combine with this approach.

The most important features selected in our models were textural and clinical ones. All models selected the clinical stage as a feature to combine with radiomic ones. Shape features were more present in those based mainly on CT images. This could be partly explained by the higher resolution of CT imaging, and therefore more detailed segmentation of tumor volume and shape features.

Our study has limitations. First, we implemented only three ML algorithms. They were chosen for their popularity in the field and because they had demonstrated good performance in several studies, including our own previous investigations in a subset of the cohort used in the present study. In addition, they were implemented with embedded feature selection, which avoids the need to study numerous combinations of feature-selection methods and classifiers, as previously conducted [9,19]. Their hyper parameters had previously been optimized, so the results presented here are the best we could obtain with these approaches. Second, although we performed prospective evaluation, in contrast to most existing radiomic studies, which have been retrospective only, our testing cohort remains small (51 patients). As a result, the differences in performance between the methods are not highly significant, in the sense that they lead to a small number of patients being classified differently. For instance, regarding the median OS endpoint, 32 patients out of 51 are correctly classified for the worst result using LR, a number that rises to 40 with the best fusion strategy. For the OS < 6 months endpoint, this ranges from 38 to 45. The hierarchy between the ML algorithms and the improvement provided by their consensus should, therefore, be considered as trends only, to be further validated in future investigations using a larger cohort, which is currently still under recruitment.

Another limitation is that we focused on binary classification tasks only, which means that when a patient with an OS barely above (or below) the median value (or the 6 months threshold) is considered for the purpose of training, the model is exactly the same as a patient with much shorter (or longer) OS. Our future investigations will thus include time-to-event modeling evaluated with appropriate metrics such as the C-index (Uno, et al. 2011). In order to partly alleviate this limitation, we included two different endpoints (median and 6 month OS). Interestingly, the performance of the models was higher for the more clinically useful second endpoint of identifying the smaller subset of patients with poor prognosis (OS < 6 months), with accuracy in the testing set ranging from 75% (RF) to 89% (fusion through averaging of probabilities), compared to 63–78% for the median OS endpoint. Finally, our study did not aim to develop and propose a fully validated prognostic model that could be picked up by the radiomics and clinical community for direct translational use in clinical practice. Our humbler goal was to investigate whether combining the outputs of several popular and established ML algorithms to train radiomics models could improve the final performance. The modest increase in accuracy is obtained at the additional cost of implementing and training several models. This cost is not an issue when using the models in practice (applying such a developed model to a new patient for which the features are available is almost instantaneous), only when training and designing them. However, one obvious limitation of such an approach is the reduced interpretability of the final prediction, since, in order to decipher the final decision, one has to understand each model (which features are being combined and how) and then how their consensus is reached. This needs to be taken into account for the future acceptability of any decision-aid tool that would be developed based on the combination of several models. Moreover, this is to be framed in the more general context of developing prognostic models [21,22,23]. The availability of data and details of the models are important prerequisites for the future development of radiomics and artificial intelligence-based prognostic models, their further validation, acceptance and eventual use in clinical practice. Our present development is only a preliminary evaluation of the potential benefit of combining several ML algorithms to achieve improved performance. Our future work will evaluate whether this finding is validated in a larger population (prospective recruitment is still ongoing) of about 200 patients, followed by a validation of the developed fused model in several external cohorts. If these future studies confirm our present preliminary findings, the final developed model and the associated data will be fully described and made available following TRIPOD and FAIR guidelines [24].

## 5. Conclusions

As shown previously in several studies, different machine learning pipelines lead to different models with different performances in stratifying patients for outcome. This was observed for both endpoints (either median overall survival or poor prognosis, with OS below 6 months). In both cases, a consensus of the different models improved the prognostic performance. Averaging probabilities achieved better improvement than majority voting in both scenarios. These preliminary findings should now be validated in a larger population.

## Figures and Tables

**Table 1:** Patients’ characteristics.

	Characteristic	No. of Patients (N = 138)	Training/Validation Set (N = 87)	Testing Set (N = 51)
Gender	Male	106 (77%)	62 (71%)	44 (86%)
	Female	32 (23%)	25 (29%)	7 (14%)
Age (y)	Range	46–94	46–94	46–89
	Mean ± SD	71.4 ± 9.4	71.4 ± 9.4	71.6 ± 10.0
Treatment	Radiotherapy only	68 (49%)	30 (34%)	28 (55%)
	Chemoradiotherapy	70 (51%)	57 (66%)	23 (45%)
Clinical stage	I	0	0	0
	II	43 (31%)	26 (30%)	17 (33%)
	III	95 (69%)	61 (70%)	34 (67%)
	IV	0	0	0

**Table 2:** Performance of the various ML methods and their consensus.

Classification	Accuracy (Median OS) (%)			Balanced Accuracy (OS < 6 Months) (%)		
	Training	Testing	# of Patients Correctly Classified	Training	Testing	# of Patients Correctly Classified
Stage 2 vs. 3	61 [95% CI 53–69]	58 [95% CI 50–66]	30	59 [95% CI 51–57]	53 [95% CI 45–61]	27
RF	89 [95% CI 84–94]	67 [95% CI 59–75]	34	100 [95% CI 100–100]	80 [95% CI 73–87]	41
SVM	100 [95% CI 100–100]	64 [95% CI 56–72]	33	92 [95% CI 87–97]	75 [95% CI 68–82]	38
LR	72 [95% CI 65–79]	63 [95% CI 55–71]	32	84 [95% CI 78–90]	78 [95% CI 71–85]	40
Fusion (majority voting)	100 [95% CI 100–100]	71 [95% CI 63–79]	36	100 [95% CI 100–100]	84 [95% CI 78–90]	43
Fusion (average of probabilities)	100 [95% CI 100–100]	78 [95% CI 71–85]	40	100 [95% CI 100–100]	89 [95% CI 84–94]	45

## Data Availability

When the prospective collection of data will be over, the images for the entire cohort (including these exploited in the present work) will be made available for research purposes, preferably on the TCIA database and following FAIR guidelines.
